# Application of construction site management based on digital twin and point cloud semantic segmentation technology

**DOI:** 10.1371/journal.pone.0340274

**Published:** 2026-03-16

**Authors:** Wenli Qin

**Affiliations:** Southwest Petroleum University-Nanchong Campus, Sichuan, China; Penn State University: The Pennsylvania State University, UNITED STATES OF AMERICA

## Abstract

Construction sites are particularly susceptible to the effects of extreme weather, with unsafe items posing a significant risk of causing substantial damage to construction projects and neighboring communities. Furthermore, data regarding the materials, machinery, and buildings present at the site are frequently obtained through manual inspection or on-site photography before the advent of extreme weather conditions. This process is resource-intensive and time-consuming. The core innovation of this study lies in the integration of digital twin technology with RandLA-Net-based point cloud semantic segmentation, optimized specifically for construction site safety management under extreme weather conditions. To achieve systematic disaster preparedness for construction sites, this study explores the potential of utilizing three-dimensional (3D) point cloud technology in construction site management. This involves acquiring location information about materials and machinery on construction sites through the development of a construction site point cloud identification system. This system is designed to identify and analyze potential risk factors in the digital twin model of a construction site, thereby optimizing the site layout at all stages. Furthermore, it enables practitioners to rapidly identify, locate, and assess potential risk factors on-site, facilitating the prompt and effective implementation of measures to prevent extreme weather.

## 1. Introduction

Dynamic and complex construction sites, including incomplete structures and uncertain resources, are among the most vulnerable environments to extreme events (e.g., hurricanes, heavy rains). Severe damage can lead to project delays and negative impacts on neighboring infrastructure (e.g., roads, power grids), significantly reducing project efficiency and causing economic losses. In the past, monitoring of construction sites was often done manually or by taking field photographs for identification and tracking purposes. However, with the advent of Building Information Modeling (BIM) technology, the construction of BIM 3D models for site management has become a hot application area.

A key feature of BIM is the 3D model, often referred to as the BIM model, which is implemented through object-oriented software [1]. The BIM model acts as a rich repository of data containing information about the geometric model and functional aspects of the constructed asset, as well as other relevant data [2], such as schedules (Progress: 4D) [3], cost estimation [4], asset management, etc [5]. It can integrate multidisciplinary information from different project lifecycle stages to facilitate communication [6]. If properly developed and managed, BIM models can provide a large amount of geometrically accurate information, as well as descriptive and actionable metadata, and be used to enhance project delivery practices [7]. However, BIM models are limited in providing real-time dynamic data of the physical environment. Building projects and assets are implemented in dynamic physical environments, generating large amounts of non-geometric data. Much of this data is underutilized but critical for informed decision-making. Therefore, this data must be collected in real-time by monitoring assets and projects. In addition, BIM models have a limited ability to handle large amounts of dynamic and multiform data that require advanced storage and processing techniques. These limitations of BIM models can lead to under-utilization of data, inefficient decision-making, and inefficient practices with significant cost implications. The emerging concept of digital twins provides a way to break the limitations of BIM. Digital twins utilize advanced data analytics, including artificial intelligence, to process large amounts of data for condition monitoring, prediction, diagnosis, forecasting, and system optimization [8]. These digital twin capabilities have the potential to significantly enhance information management and decision-making in various construction practices, thereby increasing the efficiency of construction and asset management activities.

With the latest developments in deep learning techniques for point cloud semantic segmentation and analysis, significant potential is demonstrated in streamlining the management tasks of practitioners in construction projects [9–12]. Exploring point cloud semantic segmentation and analysis will provide critical information about potentially threatening privacy and its location during extreme weather, enabling practitioners such as safety directors and supervisors to prepare more effectively and implement emergency operating procedures to protect construction sites before extreme weather events occur.

Therefore, this study applies the digital twin concept to reconstruct the reality of a construction site through point cloud inverse reconstruction and registers its information in a 3D model for safety pre-control using the point cloud semantic segmentation technique. This paper proposes a novel deep learning-based framework for analyzing construction sites. By utilizing visual point cloud data from the site field, we reconstruct a digital twin model of the construction environment, which identifies the types and locations of various objects on-site at the 3D level. This model projects semantic values onto the point cloud model based on the results of 3D semantic segmentation, thereby obtaining semantic information at the 3D level. Finally, different colors are assigned to each object, allowing people to identify and evaluate them more easily. The study assessed the performance of the proposed method on a road and bridge construction site by identifying and categorizing different objects, which will help practitioners to effectively locate potential risks on construction sites before extreme weather arrives. Thus, it can support extreme weather-informed decision-making by providing timely alerts to practitioners, thereby enhancing the safety management of construction sites in extreme weather conditions.

## 2. Literature review

### 2.1. Digital twin 3D reconstruction techniques

3D reconstruction is the mathematical modeling of 3D objects created in a virtual environment by computer representation and processing techniques [13]. The 3D reconstruction process is not only the basis for processing, manipulating, and analyzing the properties of objects in a computer environment, but more importantly, it is a critical process of establishing a virtual reality environment in a computer and simulating it in the virtual environment to represent the real world. Building 3D reconstruction technology utilizes the building as the reconstruction object. Firstly, it generates a 3D point cloud directly through 3D laser scanning or generates a 3D point cloud based on the triangulation principle using camera images. It manually aligns the 3D point cloud with the BIM model. Finally, it calculates the density of the 3D point cloud within the BIM elements after alignment to determine whether the BIM elements exist or not. Since it is impossible to avoid the existence of occlusion and the lack of data during the data acquisition process, the reasoning process can be complemented by the logical relationship between the construction process and the physical relationship between the construction components to assist in reasoning. Additionally, the BIM elements aligned with the 3D point cloud can be back-projected into the image to extract the texture information of different BIM elements, which can be used to support reasoning. One of the most essential purposes of 3D reconstruction is to obtain the construction progress, i.e., progress reasoning. Progress inference calculates construction progress by comparing the planned and completed models and identifying deviations between them. In the past few years, many methods for progress inference have been proposed, which draw on geometric, appearance, and relational information and can be broadly classified into four categories: 3D space occupancy based on point clouds, Two Dimensional (2D) planar projections based on point clouds, image variations based on 3D-2D projection regions, and relationships between geometric tuples.

The core steps of 3D modeling include acquiring depth images, performing 3D remodeling, and reconstructing surfaces [14]. The 3D reconstruction technique is the core computational process of 3D modeling, which stitches the 3D data from multiple viewpoints into a complete 3D object model. At this stage, 3D reconstruction techniques can be broadly categorized into three main types: generative learning models, statistical models, and discriminative learning models. The 3D reconstruction principle of these models mainly uses mathematical derivation to express the geometric information of the reconstructed target feature point [15–17]. The goal is to utilize the fundamental statistical characteristics of the 3D input data, typically including coordinates and color information, to describe the geometry of the target feature point accurately. The corresponding matching computation is then performed between multiple sets of features, and the best matching transformation process is computed by maximum likelihood region search [18]. However, target feature point feature extraction is usually limited by the ability of 3D modeling techniques to represent and generalize new data, and the latest data for target feature points is not specifically tailored to the input patterns of 3D modeling techniques. Therefore, strategies must be developed for different targets, e.g., using deep learning methods to adapt data patterns to other targets.

In recent years, deep learning (DL) methods, such as convolutional neural networks (CNNs), have been successful in 2D computer vision tasks, including object segmentation [19], object tracking, and object classification [20,21]. However, CNNs have limitations in modeling 3D data due to sparsity and viewpoint variations [22]. Thus, DL models were in vogue in the 3D domain to build further 3D descriptors (loaded with rich information from the original 3D data) [22–26]. Several popular DL-based 3D descriptors employ supervised learning to extract shape information from predefined datasets [9,24–26], and are often referred to as discriminative learning models. Early developments in global 3D feature learning for discriminative learning models, primarily focused on object classification [22,26,27], whereas other models concentrated on segmentation [24,25]. These deep learning models, also known as global descriptors, focus mainly on extracting semantic features of 3D models worldwide. Of course, global descriptors are insufficient to establish local correspondences, which are crucial for the correspondence matching task in the 3D reconstruction process. Since global descriptors cannot effectively learn local 3D features, a local 3D descriptor model based on deep learning is proposed to address the difficulty of teaching global descriptors [28].

The primary contribution of this work is a novel framework that integrates digital twin reconstruction with RandLA-Net semantic segmentation, specifically tailored for construction sites. This approach improves segmentation accuracy and computational efficiency compared to existing methods, as validated through multi-scenario testing.

### 2.2. Point cloud segmentation

Point cloud semantic segmentation refers to the process of classifying each point in a 3D scan into semantic categories (e.g., machinery, materials). 3D scene understanding, including point cloud segmentation, is an emerging research area with numerous applications, such as robotics, augmented reality, autonomous driving, and medical imaging [29]. The goal of scene understanding is to cluster the points belonging to a specific object in a point cloud model. To better handle 3D data, previous works have represented point cloud models in the context of multi-view images [30], voxels, and meshes [9]. Such methods help categorize points belonging to the target class but do not enable instance segmentation of objects within a specific class. Moreover, converting point cloud models to voxel and mesh representations may result in data loss, which can lead to poor classification performance [10,24]. In this regard, a deep learning framework called PointNet has been employed [24], utilizing point cloud data directly as the initial input. The PointNet framework was the first to address point alignments, and the extracted deep-learning descriptors are relatively robust for invariants in 3D point clouds. Despite the advantages of PointNet, one of the challenges is that its workflow can handle relatively small numbers of points (e.g., 1,024, 2,048, 4,096) due to the fixed size of the input layers of the deep neural network. For this reason, semantic segmentation and scene parsing of point cloud models containing millions of points (e.g., reconstructed scenes from large job sites) have been identified as a challenge for the PointNet architecture.

In the architectural domain, previous work on point cloud segmentation can be categorized into two main frameworks: model-driven and data-driven [31]. Model-driven segmentation of point clouds enables the classification of points based on hand-engineered cost functions (e.g., shape-fitting algorithms or region-growing workflows) [32]. In this regard, some studies have proposed a method to segment 3D point clouds of bricks in masonry walls [33], and region-growth-based algorithms have been used to segment infrastructure [34]. Later, a study was conducted on the automated thematic detection of point cloud data for safety regulation compliance [35]. Despite the advantages of these models, a lack of robustness to geometric variance and poor performance on noisy and incomplete point cloud models have been identified as limitations of model-driven point cloud segmentation frameworks [31]. On the other hand, data-driven models rely on different training datasets to classify points in a point cloud model. Some researchers have addressed the point cloud segmentation problem through supervised learning [36]. For example, the problem of detecting scaffolding in a point cloud was solved through a random forest framework [37]. The classification of structural bodies (e.g., columns, beams, and slabs) and reinforcement bars in laser scan data has been investigated using traditional classification methods, such as support vector machines (SVMs) [11,12]. Later, the segmentation and classification of construction machinery in 3D laser scanning models were addressed, utilizing synthetic training datasets to construct descriptors [12]. Despite its potential, the application of synthetic data remains challenging due to the limited ability to accurately represent the texture and geometric variance of point cloud models [38], which necessitates further research. Other studies have projected 2D semantic values onto point cloud models of material piles and construction equipment for 3D horizontal segmentation [39]. However, limited 3D segmentation accuracy is reported once 2D semantic information is projected onto the point cloud model [40]. In this regard, elevation-based criteria have been implemented to improve the performance of 3D semantic segmentation [39]. Despite the improved performance, elevation-based criteria are usually effective on flat surfaces; therefore, applying them to job sites that typically involve uneven surfaces will be challenging. While point cloud segmentation has advanced, model-driven approaches struggle with geometric variance, and data-driven methods require large annotated datasets. Our work addresses these gaps by leveraging RandLA-Net for efficient large-scale segmentation in complex construction environments.

## 3. Data collection and processing methods

The dataset consisted of approximately 1.5 million points collected from six construction site scenarios using a GeoSLAM ZEB Horizon LiDAR SLAM scanner. 3D reconstruction of a construction site first requires collecting raw data from the site to obtain information about the volume and location of buildings, machinery, materials, and other site features. 3D point cloud data is currently utilized as a 3D visualization tool and is popular within computer vision, particularly in dynamic real-world environments such as civil infrastructure inspection and construction schedule estimation [41,42]. A point cloud is a set of points usually containing 2D or 3D coordinates. However, a point cloud alone does not provide much information. It must further process and visualize objects within these points, such as piers and panels in road and bridge engineering infrastructure scenarios. For this reason, this study utilizes a handheld laser scanner to acquire point cloud data. It applies deep learning algorithms to identify the volume and location of machinery, materials, and other features, as well as to analyze the construction site.

### 3.1. Acquisition of construction site point cloud data

Unmanned Aerial Vehicle (UAV) photogrammetry and laser scanning can directly collect data in a variety of large and complex environments for inspection, precise navigation, and object recognition [8], for example, to identify structural elements such as walls, floors, and ceilings. The 3D point clouds, surface models, and orthogonal images generated from UAV images contain a wealth of information and are commonly used in outdoor environments. Meanwhile, with the development of mobile laser scanning technology, laser point clouds are widely used for their high accuracy and accessibility [20,21]. Handheld Simultaneous Localization and Mapping (SLAM) is a LiDAR scanner based on SLAM algorithms to acquire point clouds through mobile scanning quickly; this 3D laser scanning technology has been widely used in architecture to provide a large amount of information and points [41,42].

**Fig 1:**
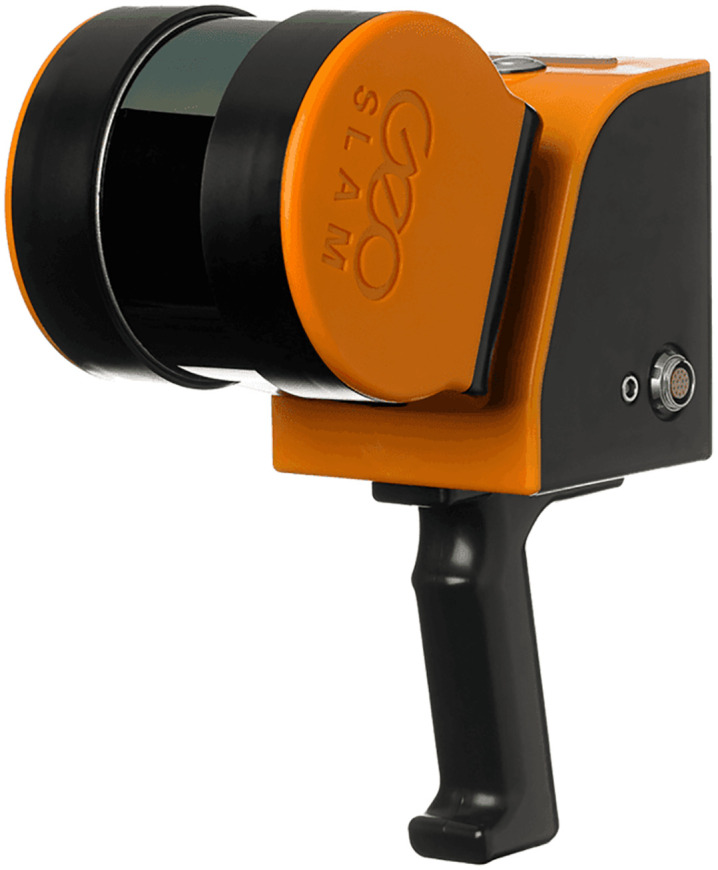
GeoSLAM ZEB horizon multi-function LiDAR scanner.

The point cloud acquisition device used in this research is the GeoSLAM ZEB Horizon multifunctional Light Detection and Ranging (LiDAR) SLAM scanner, shown in Fig 1, which can be handheld or backpack-mounted. It has a range of 100-m, can acquire 300,000 points per second, and achieves a relative accuracy of six mm.

LiDAR SLAM uses laser sensors to create a map of its environment. LiDAR stands for Light Detection and Ranging and works by sending light pulses to features and measuring the time it takes for them to reflect. This gives the exact distance of the object or feature. The output is typically 2D (x, y) or 3D (x, y, z) point cloud data and can be effectively used for 3D reconstruction.

### 3.2. RandLA-Net point cloud semantic segmentation

Due to the complexity of the construction environment, this study establishes a construction site point cloud segmentation model based on RandLA-Net. It develops and establishes a construction site point cloud recognition system using the Python programming environment. Training parameters: 100 epochs, batch size 8, cross-entropy loss function, Adam optimizer, and NVIDIA RTX 3080 GPU computational setup.

#### 3.2.1. Sampling.

In large-scale 3D point cloud recognition, it is a non-trivial task to achieve efficient semantic analysis. Existing techniques mainly rely on complex sampling techniques and pre-processing and post-processing containing heavy computation, while RandLA-Net is an efficient and lightweight technique that can be used in large point clouds by using random point sampling instead of other complex sampling techniques. Selects a certain number of points from all the points, and since it is a random selection with equal probability, the complexity is low. Its computation is not related to the total number of input point clouds but only to the number of points to be sampled, which is good in terms of real-time performance and scalability.

#### 3.2.2. Feature extraction integration.

**Fig 2:**
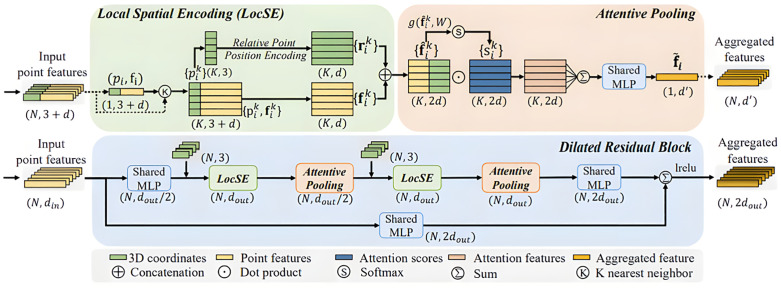
Local feature integration.

To solve the information loss caused by random sampling, RandLA-Net prevents the loss of important data by aggregating local features. As shown in Fig 2 [43]:

Local feature integration has three main parts: Local Spatial Encoding (LocSE), Attentive Pooling, and Dilated residual block.

(1) Local spatial encoding

Given a point cloud P, with all point features (e.g., raw RGB, normals, or intermediate learned features), the x-y-z coordinates of each neighborhood are embedded and displayed by the LocSE unit so that the corresponding point features can be observed, and their relative spatial locations learned. This allows the LocSE unit to observe the local geometric features of the point cloud, allowing the network to learn more complex local structures. The LocSE unit consists of the following steps.

1) Finding proximity points

Given N points, use the k-nearest neighbors (KNN) algorithm once for each point to find the K points with the closest Euclidean distance (Euclidean distance). Euclidean distance is a commonly used metric for calculating the distance between two points and measuring the similarity or dissimilarity between samples. For two n-dimensional vectors x and y, the Euclidean distance is calculated as shown in Equation 1 (*x*_*1*_*, x*_*2*_*, …, x*_*n*_ and *y*_*1*_*, y*_*2*_*, …, y*_*n*_) denote the values of each dimension of vectors *x* and *y*, respectively).


$$d(x,y)=\sqrt[{\text{2}}]{{\left(x_{\text{1}}-y_{\text{1}}\right)}^{\text{2}}+{\left((x_{\text{2}}-y_{\text{2}}\right)}^{\text{2}}+...+{\left((x_{n}-y_{n}\right)}^{\text{2}}}$$ (1)

2) Relative position coding

The green part is the local space coding, random sampling of the selected points using the KNN algorithm to obtain *K* points; each point has *3 + d* attributes, where *3* represents the position coordinates in three-dimensional space, *d* represents the feature attributes, the three-dimensional coordinates are taken out, which is the *K* three-dimensional vectors, the specific operation is for obtaining the nearest *K* points {$p_{i}^{\text{1}}\cdots p_{i}^{k}\cdots p_{i}^{K}$} for each *P*_*i*_, and encode the relative position of the points with the formula shown in Equation 2 (where $p_{i}$ and $p_{i}^{k}$ denotes the *x*, *y*, *z* coordinates of the points, *⊕* denotes stitching the dimensions together, *np.concatenation*()), which ∥∥ represents the calculation of the distance from the neighboring points to the center point).


$$r_{i}^{\;k}=MLP(p_{i}⊕p_{i}^{k}⊕(p_{i}-p_{i}^{k})⊕∥p_{i}-p_{i}^{k}∥$$ (2)

3) Point feature enhancement

Connect feature $f_{i}^{\;k}$ of each nearest neighbor point $p_{i}^{k}$ with the encoded relative point position feature $r_{i}^{\;k}$ to get the augmented feature vector ${\hat{f}}_{i}^{\;\;k}$, and finally output a new set of nearest-neighbor point features: ${\hat{\text{F}}}_{i}=\left\{{\hat{f}}_{i}^{\;k}\right\}=\left\{{\hat{f}}_{i}^{\text{1}}\cdots {\hat{f}}_{i}^{\;k}\cdots {\hat{f}}_{i}^{\;K}\right\}$ through the LocSE unit.

(2) Attention pooling

This part of the neural unit is used to aggregate the features of neighboring points. Some existing methods use maximum or average pooling to aggregate the information from neighboring points, which may result in the loss of most of the information. RandLA-Net uses the attention mechanism to learn essential features of the neighborhood adaptively. It mainly consists of the following steps.

1) Calculate the attention value

For the local feature ${\hat{F}}_{i}=\left\{{\hat{f}}_{i}^{\;k}\right\}=\left\{{\hat{f}}_{i}^{\;\text{1}}\cdots {\hat{f}}_{i}^{\;k}\cdots {\hat{f}}_{i}^{\;K}\right\}$, a model is designed with a function *g(*${\hat{f}}_{i}^{\;k}$*,W)* to learn the attention score of each feature, which consists of multilayer perceptron with softmax activation (MLP+softmax), as shown in Equation 3, where w is the weight of the shared MLP learning.


$$s_{i}^{k}=\text{g}({\hat{f}}_{i}^{\;k},\text{W})$$ (3)

2) Weighted summation

The learned attention scores can be used as a mask matrix to select important features. These features are summed up, which is calculated by the formula: ${\tilde{f}}_{i}=\sum {\mathrm{\backslash nolimits}}_{k=\text{1}}^{K}{\hat{f}}_{i}^{\;k}{\cdot s}_{i}^{k}$. Finally, the information-rich feature vector *f*_*i*_ is generated.

(3) Expansion of residual blocks

As large point clouds are gradually down sampled, it is necessary to significantly increase the perceptual domain of each point, which can preserve more geometric detail in case of point loss. Stacking multiple LocSE and attention-pooling modules by jump-joining and expanding residual blocks preserves more information.

## 4. Constructing a point cloud semantic segmentation model for the construction site location

In deep neural networks, differences in data distribution can lead to slow convergence and network training. Additionally, if the activation output is large in deep neural networks, the corresponding gradient will be small, resulting in a slow learning rate. In contrast, RandLA-Net is a new framework for semantic segmentation of large-scale point clouds, which improves the comprehension of point cloud data by using local feature integration and expanding residual modules [43]. Local feature integration involves local spatial encoding and attention pooling, where local spatial encoding establishes a local coordinate system around each point to enhance the understanding of point cloud geometry. Attentional pooling dynamically weights the features according to the importance of each point, thereby increasing the accuracy of feature extraction. The dilation residual module expands the receptive field by introducing convolutional layers with varying dilation rates, thereby enhancing the understanding of point cloud data [43]. Although RandLA-Net achieves better performance in point cloud classification and segmentation tasks, it has some drawbacks and limitations, especially when facing complex scenes such as construction sites, which are subject to the limitations of computational resources and time due to the large size of the point cloud, particularly in high-resolution scenes.

### 4.1. Data batch normalization

To speed up the network convergence and training speed, the Batch Normalisation (BN) layer is added before the data input BN layer using normalization, the data input values with different distributions are transformed into a standard normal distribution, and the activation input values fall in the region where the nonlinear function is more sensitive to the inputs, so the network outputs are reduced to obtain a more significant gradient, which speeds up the network convergence and training speed, the specific implementation of the BN layer is shown in Table 1.

**Table 1:** Pseudo-code for BN layer implementation.

**BN implementation pseudo-code**
**Input:** A small batch of *x*-values: B = {x_1_...m};
Parameters to be learned: *γ*, *β*
**Output:** {*y*_*i*_ = BN_*γ*,*β*_(*x*_*i*_)}
$\mu_{B}\leftarrow \frac{\text{1}}{m}\sum_{i=\text{1}}^{m}x_{i}$_*B*_////calculate the sample mean
$\sigma_{B}^{\text{2}}\leftarrow \frac{\text{1}}{m}\sum_{i=\text{1}}^{m}{\left(x_{i}-\mu_{B}\right)}^{\text{2}}$//calculate the sample variance
${\hat{x}}_{i}\leftarrow \frac{x_{i}-\mu_{B}}{\sqrt{\sigma_{B}^{\text{2}}+}\epsilon }$////standardize the sample data
$y_{i}\leftarrow \gamma {\hat{x}}_{i}+\beta \equiv$BN_*γ*,*β*_(*x*_*i*_)///performs translation and scaling

Secondly, the point cloud semantic segmentation model based on RandLA-Net is established. The network structure of RandLA-Net is adjusted by adding one MLP each before and after the backbone network, combining the original input, coding, decoding, and output layers, which are composed of local spatial coding, attention pooling, and extended residual blocks for extracting and integrating the local geometric and semantic features of the point cloud. The evaluation metrics of RandLA-Net, including overall accuracy, average accuracy, and average intersection and fusion ratio, are established to measure the performance and effectiveness of the semantic segmentation of point clouds.

### 4.2. Model construction

**Fig 3:**
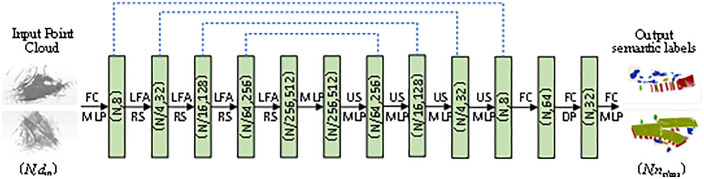
Building a semantic segmentation network architecture based on RandLA-Net (FC is Fully Connected Layer, LFA is Local Feature Aggregation, RS is Random Sampling, MLP is Shared Multi-Layer Perceptron, US is Upsampling, DP is Dropout Layer).

According to the RandLA-Net semantic segmentation principle, the overall structure is achieved by stacking multiple local feature aggregation modules and random sampling layers to accomplish the architecture of the semantic segmentation network for construction site point clouds. The features of each point of the input point cloud are first extracted using a multilayer perceptron (MLP) with standard parameters. Then, four coding and decoding layers are used to learn the features of each point. Finally, three fully connected layers and one dropout layer are used to predict the semantic labels of each point. In addition, this study incorporates one MLP before and after the backbone network to facilitate a more profound decoding mapping of input and output point clouds. The RandLA-Ne semantic segmentation network is illustrated in Fig 3. The encoder reduced point cloud size to N/256, with feature dimensions increasing from 8 to 512. Where (*N,d*) denotes the number of points and feature dimensions, respectively. Network input: The input is a large-scale point cloud of N × d_in. *N* is the number of points; *d*_*in*_ is the feature dimension of each input point. Coding Layers: Four coding layers are employed in RandLA-Net to gradually reduce the size of the point cloud while increasing the number of feature dimensions per point. Each coding layer consists of a local feature aggregation module and a random sampling component. Downsampling retains 25% of the point cloud: (*N • N/4 • N/16 • N/64 • N/256*). Meanwhile, the point-by-point feature dimension is gradually increased in each layer to retain more information: (*8 → 32 → 128 → 256 → 512*). Decoder Layers: Four decoder layers are used after the encoding layer. Each decoder layer operates as follows: 1) KNN algorithm: for each query point *Q*_*i*_, the KNN algorithm is used to find the point *P*_*i*_ that is closest to it in the input point set *I*; 2) Nearest Neighbour Interpolation: nearest neighbour interpolation is used so that the features of the query point *Q*_*i*_ are equal to the features of the closest input point *P*_*i*_; 3) Upsampling: the set of point features is up-sampled to ensure that each query point *Q*_*i*_ has a corresponding feature; 4) Jump concatenation: the up-sampled features are spliced with the intermediate features generated by the coding layer to form a new feature representation; 5) MLP application: the spliced features are processed using the MLP with shared parameters to learn the complex relationships between the features. Final semantic prediction: the final semantic labels are predicted for each point by three fully connected layers with shared parameters and a Dropout layer: (*N,64*)→(*N,32*)→(*N,n*_*class*_).

### 4.3. Evaluation metrics

The evaluation metrics include the following: Overall Accuracy (OA), Mean Accuracy (mAcc), and Mean Intersection-over-Union (mIoU): their respective Equation are shown in 4–6:


$$OA=\frac{\sum_{i=\text{1}}^{c}TP}{\sum_{i=\text{1}}^{c}\left(TP+FN\right)}$$ (4)

$$\text{mAcc}=\frac{\text{1}}{\text{c}}\cdot \sum_{\text{i}=\text{1}}^{\text{c}}\frac{\text{TP}}{\text{TP}+\text{FN}}$$ (5)

$$\text{mIoU}=\frac{\text{1}}{\text{c}}\sum_{\text{I}=\text{1}}^{\text{C}}\frac{\text{TP}}{\text{TP}+\text{FN}+\text{FP}}$$ (6)

## 5. Design of point cloud recognition system for construction site

**Fig 4:**
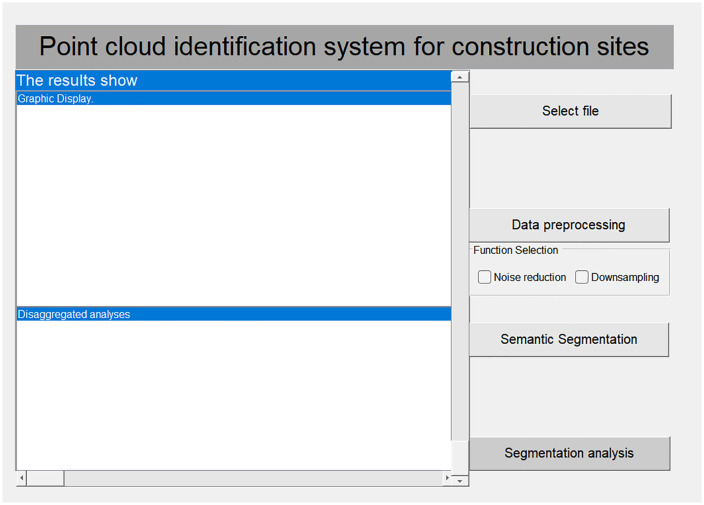
Visual interface of the system.

This study uses Python Tkinter to design a point cloud recognition system for a construction site. The design primarily utilizes controls such as comboboxes, text fields, and labels, and different modules are constructed by invoking the code. The point cloud recognition system developed in this study includes a data pre-processing module, a semantic segmentation module, and a classification and analysis module. The system’s visual interface is shown in Fig 4.

### 5.1. Data preprocessing module

The data pre-processing in this system uses a voxel grid filter to perform noise removal, filtering, and downsampling of the data. The voxel grid filter is a commonly used method for 3D data processing that can perform noise removal, filtering, and downsampling operations. Its principle is as follows:

1) Gridding: The 3D space is divided into a series of small voxel grids, each representing one voxel.
2) Data mapping: The input 3D data is mapped to the corresponding voxel grid. Typically, the data is mapped according to the spatial relationship between the data points and the grid.
3) Noise removal: For each data point within the grid, the value representing the grid is calculated by a specific algorithm (e.g., mean, median, etc.), which removes the noise from the data.
4) Filtering: information transfer and smoothing between the grids to better preserve the characteristics of the data and reduce the effects of noise. Commonly used methods include Gaussian filtering, mean filtering, etc.
5) Downsampling: Depending on the sampling rate, select a part of the grid as the output, reducing the amount of data while retaining the main features. This is usually done by merging or selecting the grids.

The advantage of a voxel grid filter is that it can process data uniformly in three-dimensional space, effectively removing noise, smoothing the data, and reducing the data size. It is widely used in computer graphics, computer vision, and other fields for 3D point cloud data processing, model reconstruction, voxelization, and other tasks.

**Fig 5:**
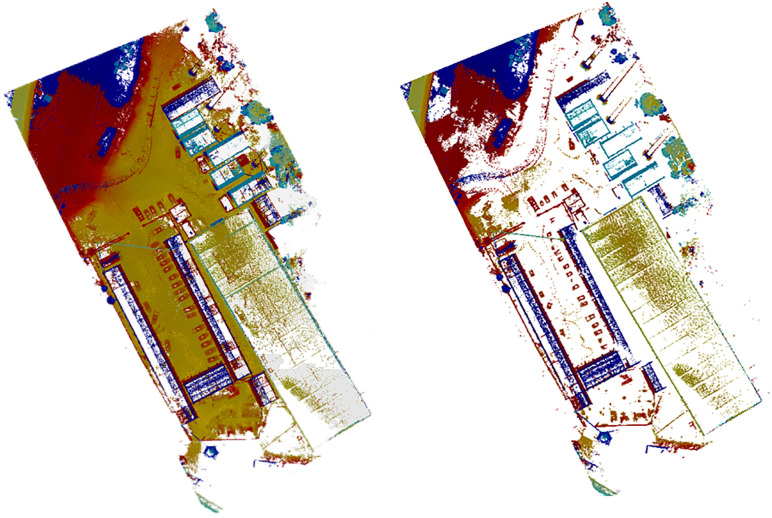
Point cloud data before and after preprocessing. (a) Pre-processed point cloud data. (b) Point cloud data after pre-processing.

This system implements the voxel grid filter using the Point Cloud (PCL) library. This module loads a point cloud data file (input_cloud.pcd) and creates a voxel grid filter object. The voxel size (1 cm) is set using the setLeafSize method. Finally, a filtering operation is performed by calling the filter method, and the result is saved as a new point cloud data file (filtered_cloud.pad). The point cloud voxel grid filter preprocesses the data part of the code, and the point cloud data before and after preprocessing is shown in Fig 5.

### 5.2. Semantic segmentation module

The construction site point cloud semantic segmentation module is developed based on the RandLA-Net model. Its function is mainly to semantically segment the pre-processed point cloud data, enter semantic segmentation labels for the different types of objects in the data, and extract the features of the point cloud data at the same time.

**Fig 6:**
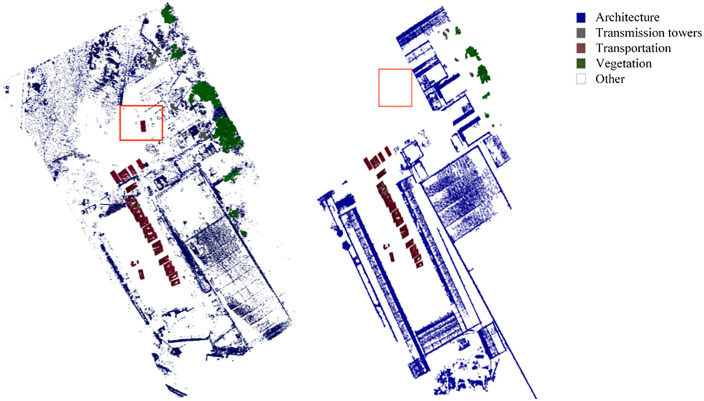
Before and after comparison of the final visualization results of the semantic segmentation module. (a) Trimble Realworks manual semantic annotationTrimble Realworks. (b) Semantic segmentation results.

This module creates datasets (large point cloud datasets for semantic segmentation tasks) and uses the point cloud classification function in Trimble Realworks to train them. After being processed by the data pre-processing module, the Training and Test (TS) dataset is imported into the point cloud semantic segmentation module for module training. Then, after the training dataset is completed, the validation dataset is subjected to data preprocessing. Finally, the semantic labels are manually added to verify the effectiveness of the point cloud semantic segmentation module. The final visualization of the trained semantic segmentation module is shown in Fig 6.

According to the visualization results and metrics evaluation, the semantic segmentation effect of the point cloud semantic segmentation module performs well. According to the results in Table 2, both OA and mAcc are at a high level, but the results of mIoU are relatively low. The main reason for this is that the number of semantic labels added in the training set is high, while the point cloud data in the validation set lacks some instances of semantic labels, which leads to a low mIoU. mIoU is not used as an evaluation index for the semantic segmentation module because it is greatly affected by the different types of point cloud scenes. In addition, it can be seen from the visualization result display that there is an unrecognized transport vehicle (marked in red). In addition, the detection range of the vegetation during the detection process is slightly reduced compared to the actual labels, but the number of detections does not have an error. The reason for this type of error is, on the one hand, the accuracy of the equipment, and, on the other hand, the point cloud is sparse and removed during the recognition process, which slightly impacts the recognition results. However, from the overall performance of the semantic segmentation module in the dataset, the integrated effect of constructing the point cloud semantic segmentation model based on RandLA-Net is good, and it can be used in the scene semantic segmentation module of the point cloud recognition system. However, adding a relatively large number of semantic labels for training in combination with the construction site in the later practical application process is necessary.

**Table 2:** Running effect of point cloud semantic segmentation module.

Index	OA (%)	MAcc (%)	MIoU (%)
**Fruit**	94.2	84.6	60.3

### 5.3. Classification analysis module

This module mainly detects whether the layout of the construction site is reasonably distributed during the construction process, such as the location of components and the distribution of materials, through the point cloud data after semantic segmentation. The classification analysis is performed by constructing a KD tree of the construction model to find the nearest neighbor points quickly. Then, each point of the point cloud is traversed, and the nearest neighbor is searched using the KD tree to calculate the closest distance of each point on the design model. Next, a new ColorMap representation is created for classification based on the normalized value of the distance. Finally, the color classification is applied to the point cloud data, and the construction site distribution is displayed using Open3D’s visualization feature.

## 6. Case validation

### 6.1. Construction site data collection and pre-processing

**Fig 7:**
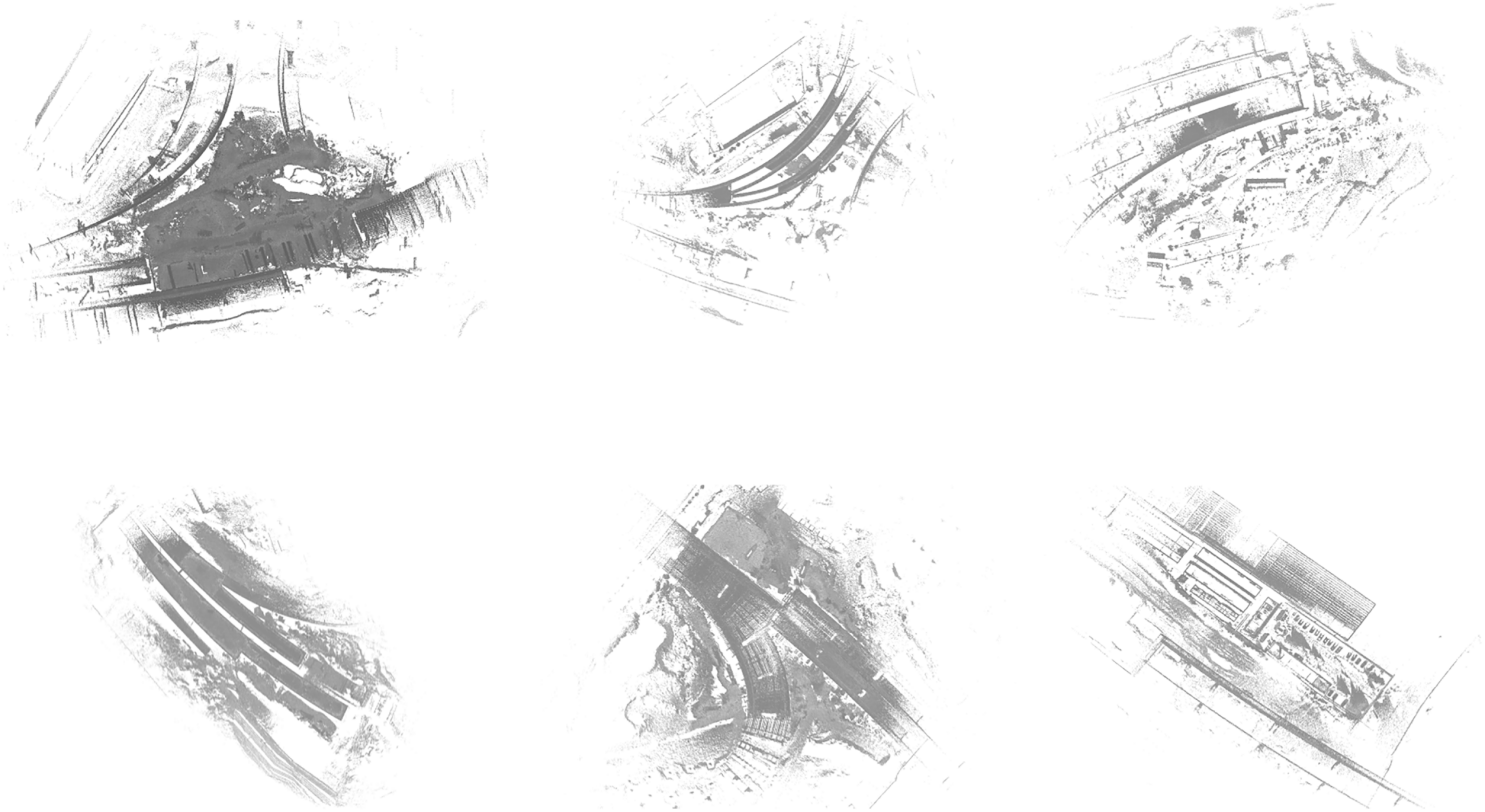
Original point cloud model.

This study was evaluated in terms of classification effectiveness and accuracy to validate the reliability of the point cloud recognition system at the construction site. The point cloud data used to validate the system’s reliability comes from a part of the construction site of an overpass section of a project, collected using GeoSLAM ZEB; the raw point cloud data collected and created in the experimental area is shown in Fig 7. The data acquisition process is based on the construction site, as the device (GeoSLAM ZEB) acquisition takes 20 minutes for the path to close the loop, so a sub-area scan was taken for data acquisition.

**Fig 8:**
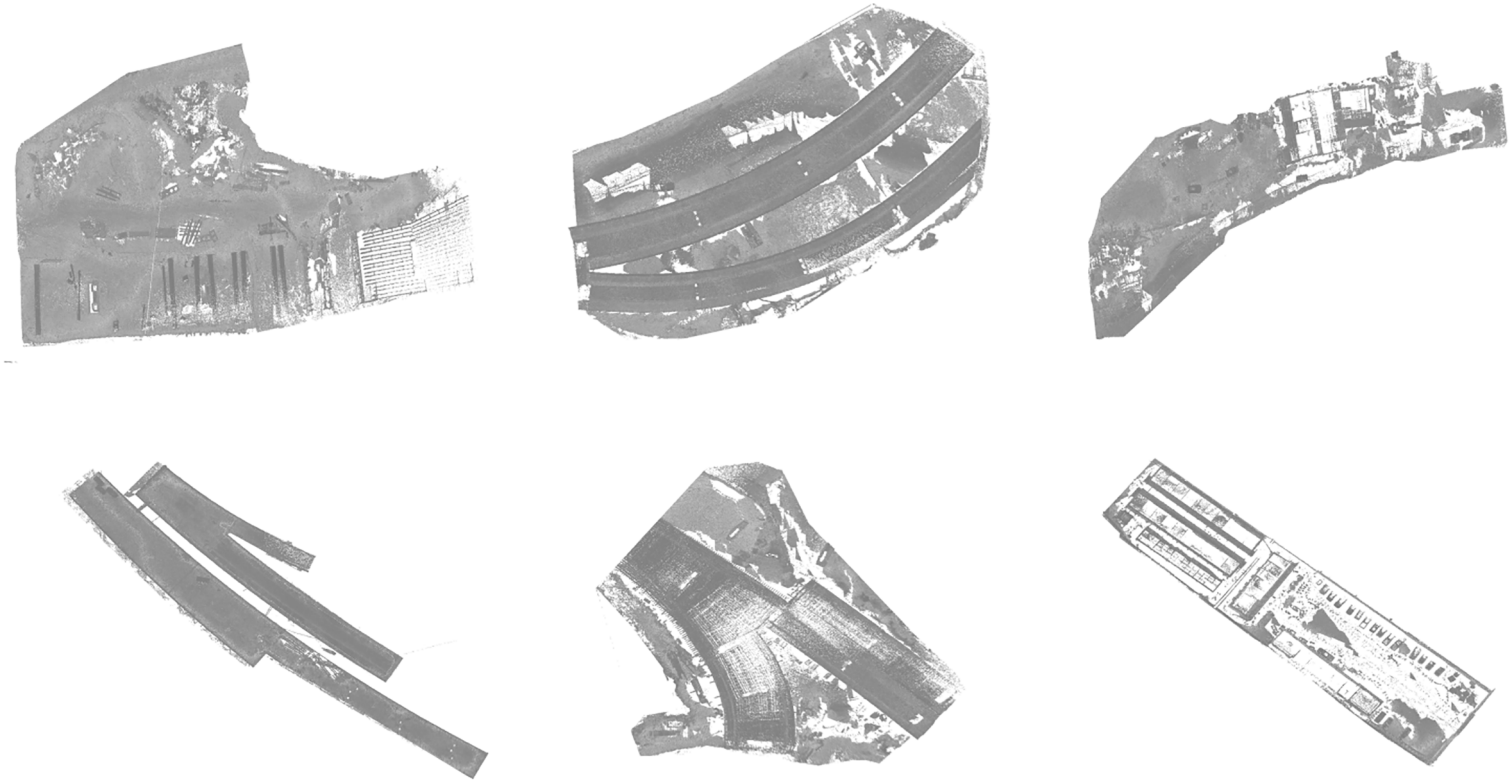
Preprocessed point cloud model.

The data preprocessing module removes the large amount of noise contained in the acquisition process of the point cloud data, and the processed point cloud model is shown in Fig 8.

### 6.2. Point cloud semantic segmentation results and analysis

**Fig 9:**
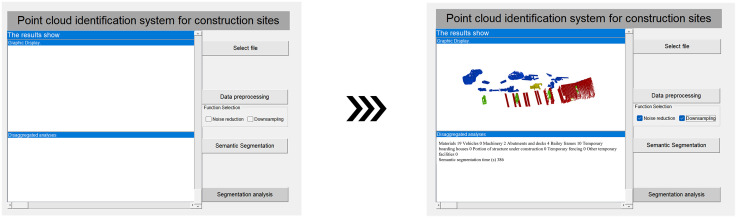
Point cloud semantic segmentation process.

To perform semantic segmentation on the pre-processed point cloud, click on the semantic segmentation module to start semantic segmentation of the input point cloud model, and the system displays the semantic segmentation results and statistical results of the point cloud through classification and analysis in the graphical display interface and text description interface on the left side of the system after the segmentation is completed, as shown in Fig 9.

**Fig 10:**
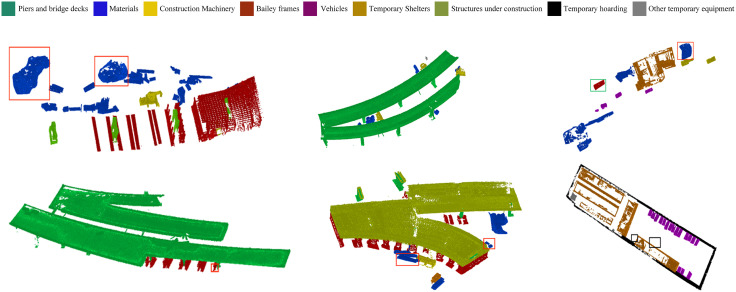
Display of point cloud semantic segmentation results.

The point cloud semantic segmentation results are shown in Fig 10, and the statistical results are in Table 3. From the figure, it can be seen that the overall recognition effect of the semantic segmentation results of the six regions is good, which can identify and segment the types of completed piers and bridge decks, materials, construction machinery, and berths in the captured region. However, there are, for example, errors in the recognition of materials (marked by red rectangles in Fig 10 regions 1, 3, and 5), errors in the recognition of berths (marked by green rectangles in Fig 10 regions 3 and 4) and omissions in the recognition of vehicles (marked by black rectangles in Fig 10 region 6). The final accuracies were calculated according to the Overall Accuracy (OA) formula, as shown in Table 3, which is a decrease compared to the overall accuracy during training. Table 3 shows that the accuracy of materials, vehicles, and beribboned frames is less accurate than the other types of detections. On the other hand, the accuracy is higher for the categories with an accuracy of 1 due to the small number of their categories and their distinctive features. This is because the piles of stones in areas 1 and 5 and some rubbish in area 3 are identified as materials, and the materials in areas 3 and 4 are incorrectly identified as bailey racks. The error may be because the similarity of the features in the point cloud is too high, causing the model to classify the point cloud with a high degree of similarity into the same category in the semantic segmentation process. Subsequently, for the model to perform this category of features for targeted training, the training process needs to extract more feature points to increase the classification accuracy. For the recognition error of the vehicles in Region 6, according to the analysis, the error is due to three reasons: 1) the two regions were missed due to the proximity of the point cloud and other categories and features are not obvious; 2) the use of data acquisition instrument ZEB-REVO precision is low; the acquisition process of the route planning problems caused by the features are not obvious. The solutions to these three types of problems are: 1) to obtain good data by optimizing the acquisition path according to the best acquisition distance and angle of the instrument; 2) to use a higher precision instrument with path optimization for data acquisition. In addition, the detection time of the semantic segmentation module will vary according to the number of categories and the size of the detection area. However, in the end, the semantic segmentation of the point cloud model will be completed in between 150 and 400 seconds for an acquisition area. The study evaluated six site areas (Fig 10). Compared to PointNet, our method achieved 10% higher mIoU. Computational time averaged 300 seconds per area. The confusion matrix (Table 4) reveals misclassification between materials and Bailey frames.

**Table 3:** Statistics of semantic segmentation results (Note: types of recognition errors in parentheses).

Form	Test area						Overall Accuracy
	1	2	3	4	5	6	
Materials	19 (2)	5	5 (1)	0	5 (2)	0	0.852
Vehicles	0	2	4	0	0	17 (2)	0.913
Machinery	2	3	2	0	0	0	1
Piers and decks	4	9	0	21	12	0	1
Bailey frames	10	0	(1)	5 (1)	16	0	0.938
Temporary Shelters	0	0	1	0	0	5	1
Structures under construction	0	0	0	0	1	0	1
Temporary hoarding	0	0	0	0	0	1	1
Other temporary facilities	0	1	0	0	1	1	1
Semantic segmentation time (s)	386	214	151	206	241	285	

**Table 4:** Confusion matrix for semantic segmentation results.

Actual Predicted	Materials	Vehicles	Machinery	Piers and Decks	Bailey Frames	Temporary Shelters	Other Facilities	Recall
Materials	32	1	0	0	3	0	0	88.9%
Vehicles	0	21	0	0	0	0	2	91.3%
Machinery	0	0	7	0	0	0	0	100%
Piers and Decks	0	0	0	46	0	0	0	100%
Bailey Frames	2	0	0	0	29	0	0	93.5%
Temp. Shelters	0	0	0	0	0	5	0	100%
Other Facilities	0	1	0	0	0	0	8	88.9%
Precision	94.1%	91.3%	100%	100%	90.6%	100%	80.0%	

## 7. Conclusion and discussion

### 7.1. Conclusion

At present, the site lacks intelligent monitoring and management technology. It mainly relies on manual inspection, but there are problems with high subjectivity, low efficiency, and susceptibility to omission. Deep learning and 3D point cloud semantic segmentation technology has recently started to be applied to construction site safety progress inspection, but it is still in the exploration stage. This study explores the application of 3D point cloud technology in construction site management and analyzes how to quickly and accurately capture the point cloud data of a construction site from the perspective of engineering practice to optimize the site’s layout at each stage. Automated visual management was ultimately realized. This study provides a practical framework for automated risk assessment, reducing reliance on manual inspection. These results are of great significance to the development of the building construction industry. From the point of view of engineering practice, the fast and accurate acquisition of point cloud data from a construction site, and then the classification of the point cloud by a construction site point cloud recognition system, helps to optimize the site layout at all stages. This method makes seeing the difference between the physical object and the model more intuitive. It can comprehensively consider the transformation of the site at different stages, combine with the concept of saving land in green construction, avoid redundancy of land use, minimize the occupation of construction land, make the layout compact and reasonable, and at the same time meet the relevant requirements of fire safety and civilized construction. In addition, this technology can quickly identify, locate, and assess potential risk factors in construction sites for effective and timely management. To this end, the work done in this paper and its significance are as follows:

(1) Acquiring point cloud data through on-site 3D laser scanning technology and identifying and classifying the point cloud can help provide clues for construction personnel to quickly identify, locate, and assess potential risk factors in a construction site to implement management effectively and timely.
(2) Point cloud data shows the state of the construction site to facilitate the optimization of the site layout at various stages. For example, comprehensive consideration of site transformation at multiple stages, combined with the concept of saving land in green construction to avoid redundancy of land, visual display of the land situation to minimize the occupation of construction land so that the layout is compact and reasonable, and at the same time to achieve the site appearance of neat, smooth roads, by fire safety and civilized construction and other related requirements.

Overall, in the digital twin environment, the construction site will be registered in the 3D model through the point cloud information for pre-control safety production to ensure safety production and more convenient management during the construction process.

### 7.2. Discussion

This study uses the digital twin concept to classify and visualize the construction site reality through point cloud semantic segmentation technology for safety production pre-control, which can control the construction site, but there are still some shortcomings.

(1) In this study, a handheld laser scanner is used to collect point cloud data, which is inefficient, has a small coverage area, and limited accuracy. Future research can consider using a laser scanner with higher accuracy to collect point cloud data quickly at the construction site and combining the optimal collection distance and angle of the instrument with the optimization of the collection path to obtain good data, thereby improving the efficiency and quality of data collection.
(2) This study’s semantic segmentation results are affected by the similarity of point cloud features, and there are recognition errors and omissions. Subsequently, the model carries out this category of features for targeted training in the training process to extract more feature points to increase classification accuracy.

Limitations include computational cost for large-scale scenes and accuracy issues in complex environments. Future work will focus on real-time processing and multi-sensor fusion.

## Supporting information

S1 File Item Description: RandLANet.py is the semantic segmentation code for the paper. (PY)
